# Monkeypox Virus in Healthcare Settings, Sierra Leone, June 2025

**DOI:** 10.3201/eid3210.251697

**Published:** 2026-10

**Authors:** Michaella Jaba, Emmanuel Saidu, Emmanuel S. Kamanda, Christina Frederick, Laurens Liesenborghs, Megan Halbrook, Nicole A. Hoff, Placide Mbala-Kingebeni, Isaac I. Bogoch, Souradet Y. Shaw, Anne W. Rimoin, Jia B. Kangbai, Jason Kindrachuk

**Affiliations:** University of Manitoba, Winnipeg, Manitoba, Canada (M. Jaba, C. Frederick, S.Y. Shaw, J. Kindrachuk); Njala University, Bo, Sierra Leone (E. Saidu, E.S. Kamanda, J.B. Kangbai); Institute of Tropical Medicine, Antwerp, Belgium (L. Liesenborghs); KU Leuven, Leuven, Belgium (L. Liesenborghs); University of California, Los Angeles, California, USA (M. Halbrook, N.A. Hoff, A.W. Rimoin); National Institute for Biomedical Research, Kinshasa, Democratic Republic of the Congo (P. Mbala-Kingebeni); Toronto General Hospital, University Health Network, Toronto, Ontario, Canada (I.I. Bogoch); Public Health Agency of Canada, Ottawa, Ontario, Canada (J. Kindrachuk)

**Keywords:** Mpox, monkeypox virus, viruses, zoonoses, sexually transmitted infections, Sierra Leone

## Abstract

During the 2025 mpox outbreak in Sierra Leone, we assessed environmental contamination in 2 hospitals. Of 89 surfaces sampled, 6 (6.7%) surface samples, primarily from doors, were PCR-positive for monkeypox virus. Our findings highlight monkeypox virus surface contamination in healthcare settings and help prioritize infection prevention and control targets in resource-limited settings.

Mpox, caused by monkeypox virus (MPXV), is a zoonotic disease historically endemic to Central and West Africa (*1*). Since 2022, the global spread of MPXV clade IIb has altered transmission dynamics, and reports of sustained human-to-human transmission have increased (*2*,*3*). Environmental contamination by MPXV has been identified, including recovery of infectious virus particles in healthcare facilities where infection prevention and control (IPC) resources might be limited (*4*,*5*). Beginning in January 2025, Sierra Leone experienced a substantial mpox outbreak, representing one of the country’s largest recorded clusters, which strained public health resources. Data on environmental contamination in healthcare settings in Africa during active mpox outbreaks remain scarce. We conducted surface sampling in 2 major urban hospitals in Sierra Leone to assess the presence of MPXV and identify high-risk contamination sites to inform local IPC strategies.

## The Study

The study was conducted in June 2025 at Connaught Hospital in Freetown, the capital and largest city of Sierra Leone, and Bo Government Hospital in Bo, Sierra Leone. Both facilities are referral centers currently managing suspected and confirmed mpox cases during the national mpox outbreak. We sampled 89 high-touch surfaces in clinical and nonclinical areas, including patient beds, door handles, bathroom fixtures such as toilet seats and faucet taps, and nonsingle-use medical equipment such as medical instrument trays and blood pressure cuffs that are supposed to be sterilized after every use. We swabbed each surface for ≈3 minutes by using a polyester-tipped applicator premoistened with viral transport media, by following a standardized protocol (*6*). We standardized surface area swabbing by using a template. Our sampling did not target specific rooms with known mpox patients and was conducted without interfering with routine cleaning schedules, to reflect typical environmental conditions. The study was approved by the Sierra Leone Ethics and Scientific Review Committee (protocol no. 010/05/2025) and the University of Manitoba Health Research Ethics Board.

We extracted DNA by using KingFisher Flex (Thermo Fisher Scientific, https://www.thermofisher.com), a robotic magnetic bead–based system. We performed real-time PCR targeting the MPXV B6R gene by using published primers and probe sequences (*7*). The 40-cycle assay included negative and positive controls. We set thermal cycling conditions to 95°C for 2 minutes, followed by 40 cycles of 95°C for 15 seconds, and 60°C for 1 minute. We established a positive cycle threshold (Ct) cutoff of <38 on the basis of other environmental MPXV studies (*8*).

Of the 89 surfaces sampled, 6 (6.7%) surface samples were PCR-positive for MPXV. Positivity was 4.0% in Freetown (2 positive of 50 tested) and 10.3% in Bo (4 positive of 39 tested); this difference might reflect variation in IPC practices or frequency of mpox patient contact with sampled surfaces. Ct values for positive samples ranged from 32.34–37.91 (Appendix Table), indicating a range of viral load. A total of 28 doors were sampled and accounted for 2 (33.3%) of the 6 positive surfaces. Other positive surfaces included a delivery bed and weighing scale, a medical ward instrument tray, a laboratory, an office bathroom, and an eye clinic table (Appendix Table). The concentration of MPXV DNA on doors and other high-touch points aligns with fomite transmission risks identified in similar investigations (*9*).

Although Ct values are not a direct proxy for infectivity, lower Ct values (<30) might indicate a higher likelihood of viable virus (*8*). The positive Ct values we observed suggest moderate viral DNA load on those surfaces, likely below the threshold for recovering infectious viruses. Other studies report similar findings, in which Ct values ranged from 22–38 on high-touch objects in healthcare and residential settings (*10*,*11*).

## Conclusions

We detected MPXV DNA on 6.7% of sampled high-touch surfaces in 2 hospitals in Sierra Leone during the 2025 national mpox outbreak. Doors were the most contaminated surface type, consistent with studies from other settings and highlighting their role as critical fomites requiring targeted disinfection (*10*,*12*). The Ct values observed from positive samples (mostly >32) indicate the presence of MPXV DNA, but do not suggest infectivity. However, we did not perform viability studies. Although culture-based studies suggest a viable virus is less likely at Ct values >30, the detection of the MPXV DNA on high-contact surfaces underscores their potential as a reservoir for transmission in the absence of consistent, low, or proper IPC practices (*13*). Our study suggests that doors and other high-touch surfaces within healthcare clinics assessed are highly susceptible surfaces for MPXV contamination. This could reflect the large number of patients entering and leaving various rooms within the healthcare facility and the low frequency or improper practice of IPC within the hospital.

Limitations of our study include the cross-sectional design and purposive sampling of specific high-touch surfaces, which limits generalizability, and a lack of data on cleaning frequency or direct links to nosocomial transmission events. Furthermore, the epidemiologic context of the 2025 mpox outbreak in Sierra Leone, with high community transmission, likely influenced the background level of environmental contamination within healthcare facilities.

Despite those limitations, detection of MPXV DNA on high-touch surfaces in a resource-constrained setting reinforces the necessity of robust IPC measures and considerations. The 2025 mpox outbreak in Sierra Leone underscores the ongoing threat of mpox in West Africa and the strain on health systems. Strengthened environmental hygiene, focused and frequent disinfection of high-touch surfaces such as doors and bed rails, and adherence to hand hygiene, are necessary to reduce surface contaminations in healthcare settings (*14*). Integrating simple, periodic environmental surveillance into outbreak response might help identify IPC gaps, guide resource allocation, and tailor staff training in similar low-resource settings (*15*).

Appendix Additional information about monkeypox virus in healthcare settings, Sierra Leone, June 2025.
