# Supplementary material for: Monkeypox Virus in Healthcare Settings, Sierra Leone, June 2025
Source: Emerg Infect Dis. 2026 Oct;32(10):1699–701. doi: 10.3201/eid3210.251697 (PMC13632870; doi:10.3201/eid3210.251697)
Supplement: Supplementary file 1 [file 25-1697-Techapp-s1.pdf]

Article DOI: https://doi.org/10.3201/eid3210.251697

EID cannot ensure accessibility for supplementary materials supplied by authors. Readers who have difficulty accessing supplementary content should contact the authors for assistance.

Monkeypox Virus in Healthcare Settings, Sierra Leone, June 2025

Appendix

Appendix Table. Summary of Cycle Threshold Values for MPXV-Positive Surface Samples, Sierra Leone, June 2025. N/A represents no PCR amplification.

Hospital Location	Surface Description	Ct Value	Ct Value Interpretation
FREETOWN	Clinical Office Door	38.19	Negative
FREETOWN	OPD Main Door	N/A	Negative
FREETOWN	Nurses Table	N/A	Negative
FREETOWN	Doctors Office Table	N/A	Negative
FREETOWN	OPD Nurses Room Desk	N/A	Negative
FREETOWN	Doctors Office	N/A	Negative
FREETOWN	Corridor	N/A	Negative
FREETOWN	Surveillance Unit Door	N/A	Negative
FREETOWN	Medical Ward Door Handle	38.69	Negative
FREETOWN	Doctors Office	N/A	Negative
FREETOWN	Triage Door	N/A	Negative
FREETOWN	Medical Ward Patient Bed	40.79	Negative
FREETOWN	Medical Ward Main Door	N/A	Negative
FREETOWN	Surveillance Unit Table Surface	39.86	Negative
FREETOWN	Medical Ward Main Door	39.2	Negative
FREETOWN	Alcohol Swab	39.3	Negative
FREETOWN	Doctors Office Door	N/A	Negative
FREETOWN	Medical Ward Door	N/A	Negative
FREETOWN	Doctors Office Nurses Table	N/A	Negative
FREETOWN	Medical Ward Nurses Table	N/A	Negative
FREETOWN	Trauma Unit Nurses Table	N/A	Negative
FREETOWN	Metal Chair	38.77	Negative
FREETOWN	Nurses Trolley	N/A	Negative
FREETOWN	Medical Ward Bathroom Door	N/A	Negative
FREETOWN	Trauma Unit Main Door	N/A	Negative
FREETOWN	Medical Ward Main Door	40.74	Negative
FREETOWN	OPD Resuscitation	N/A	Negative
FREETOWN	Triage Door	N/A	Negative
FREETOWN	OPD Nurses Toilet Door	40.91	Negative
FREETOWN	Medical Ward Corridor	N/A	Negative
FREETOWN	Medical Ward Nurses Room Door	N/A	Negative
FREETOWN	Triage Nurses Table	N/A	Negative
FREETOWN	Trauma Unit Patient Bed	N/A	Negative
FREETOWN	Medical Ward Instrument Tray	38.37	Negative
FREETOWN	Medical Ward Trolley	N/A	Negative
FREETOWN	Signing Office Door	N/A	Negative
FREETOWN	Matrons Office Main Entrance	N/A	Negative
FREETOWN	Medical Ward Toilet Door	37.91	Positive
FREETOWN	Records Door	N/A	Negative
FREETOWN	Ent Door	N/A	Negative
FREETOWN	Medical Ward Main Door	N/A	Negative
FREETOWN	Medical Ward Corridor	N/A	Negative
FREETOWN	SOP Main Table	N/A	Negative
FREETOWN	Records Table	N/A	Negative
FREETOWN	Dialysis Door	N/A	Negative
FREETOWN	Medical Ward Door Handle	38.69	Negative
FREETOWN	Senior Matron Toilet Door	N/A	Negative
FREETOWN	Medical Ward Main Door	36.92	Positive

Hospital Location	Surface Description	Ct Value	Ct Value Interpretation
FREETOWN	Blood Pressure Cuffs	38.92	Negative
FREETOWN	Nurses Table	N/A	Negative
BO	OPT	N/A	Negative
BO	HEA	N/A	Negative
BO	Medical Ward Room	N/A	Negative
BO	KMC	N/A	Negative
BO	Plebo	N/A	Negative
BO	Maternity	N/A	Negative
BO	Medical Ward Instrument Tray	36.83	Positive
BO	Chem	N/A	Negative
BO	Medical Ward Room	N/A	Negative
BO	Outpatient (Wastewater)	N/A	Negative
BO	Bacteriology	N/A	Negative
BO	Laboratory Office Bathroom	32.34	Positive
BO	Handwash Maternity	N/A	Negative
BO	Labor Ward Delivery Bed & Weighing Scale	37.83	Positive
BO	Medical Ward Patient Toilet & Treatment Table	N/A	Negative
BO	Plastic Chair & Arm Rest	39.5	Negative
BO	Main Operation Theater, Sluice Room & Operation Bed	N/A	Negative
BO	Main Handwashing Station & Entrance	N/A	Negative
BO	Isolation Unit (Wash H& Station & Entrance Pillar)	N/A	Negative
BO	Triage (Table & Examination Bed)	40.11	Negative
BO	Lactating Ward (Toilet & Wash H& Site)	N/A	Negative
BO	Maternity Operations Theater (General & Infection Waste Bin)	N/A	Negative
BO	Maternity Theater Patient & Staff	N/A	Negative
BO	High Dependency Unit (Suction & Oxygen Concentration)	N/A	Negative
BO	Main Operation Theater Dressing Room (Dressing Bed & Waste Bin)	N/A	Negative
BO	Labor Ward, Laundry Room, & Placenta Receiver	N/A	Negative
BO	Maternity Triage Unit Trolley & Staff Table	N/A	Negative
BO	Maternity Theater Main Entrance & Exit Door	N/A	Negative
BO	Neonatal Ward Examination Bed & Weighing Scale	N/A	Negative
BO	Medical Ward Theater, Body Resuscitation Bed & Anesthetic Machine	N/A	Negative
BO	Medical Ward H& Washing Site & Treatment Tray	N/A	Negative
BO	Vision Lens	39.1	Negative
BO	Medical Ward Theater Instrument Cabinet & Drawer	N/A	Negative
BO	Medical Ward Infectious & General Waste Bin	N/A	Negative
BO	Medical Ward Infectious & General Waste Bin	N/A	Negative
BO	Medical Ward Staff Table & Patient Bed	N/A	Negative
BO	Eye Clinic Vision Lens & Alcohol Swab Table	37.88	Positive
BO	Medical Ward Treatment Tray & Staff Table	36.83	Positive
BO	Maternity Theater Recovery Bed & Instrument Trolley	39.26	Negative
